# Handheld Briefcase Optical Coherence Tomography with Real-Time Machine Learning Classifier for Middle Ear Infections

**DOI:** 10.3390/bios11050143

**Published:** 2021-05-03

**Authors:** Jungeun Won, Guillermo L. Monroy, Roshan I. Dsouza, Darold R. Spillman, Jonathan McJunkin, Ryan G. Porter, Jindou Shi, Edita Aksamitiene, MaryEllen Sherwood, Lindsay Stiger, Stephen A. Boppart

**Affiliations:** 1 Department of Bioengineering, University of Illinois at Urbana-Champaign, Urbana, IL 61801, USA; jwon8@illinois.edu; 2 Beckman Institute for Advanced Science and Technology, University of Illinois at Urbana-Champaign, Urbana, IL 61801, USA; gmonroy2@illinois.edu (G.L.M.); rdsouza@illinois.edu (R.I.D.); dspillm2@illinois.edu (D.R.S.J.); jindous2@illinois.edu (J.S.); edaks@illinois.edu (E.A.); 3 Department of Otolaryngology, Carle Foundation Hospital, Champaign, IL 61822, USA; jonathan.mcjunkin@carle.com (J.M.); ryan.porter@carle.com (R.G.P.); 4 Carle Illinois College of Medicine, University of Illinois at Urbana-Champaign, Champaign, IL 61820, USA; 5 Department of Electrical and Computer Engineering, University of Illinois at Urbana-Champaign, Urbana, IL 61801, USA; 6 Stephens Family Clinical Research Institute, Carle Foundation Hospital, Urbana, IL 61801, USA; maryellen.sherwood@carle.com (M.S.); lindsay.stiger@carle.com (L.S.)

**Keywords:** biofilms, handheld, machine learning, middle ear infections, optical coherence tomography, tympanic membrane

## Abstract

A middle ear infection is a prevalent inflammatory disease most common in the pediatric population, and its financial burden remains substantial. Current diagnostic methods are highly subjective, relying on visual cues gathered by an otoscope. To address this shortcoming, optical coherence tomography (OCT) has been integrated into a handheld imaging probe. This system can non-invasively and quantitatively assess middle ear effusions and identify the presence of bacterial biofilms in the middle ear cavity during ear infections. Furthermore, the complete OCT system is housed in a standard briefcase to maximize its portability as a diagnostic device. Nonetheless, interpreting OCT images of the middle ear more often requires expertise in OCT as well as middle ear infections, making it difficult for an untrained user to operate the system as an accurate stand-alone diagnostic tool in clinical settings. Here, we present a briefcase OCT system implemented with a real-time machine learning platform for middle ear infections. A random forest-based classifier can categorize images based on the presence of middle ear effusions and biofilms. This study demonstrates that our briefcase OCT system coupled with machine learning can provide user-invariant classification results of middle ear conditions, which may greatly improve the utility of this technology for the diagnosis and management of middle ear infections.

## 1. Introduction

Otitis media (OM), commonly known as a middle ear infection, is a disease caused by bacterial and/or viral pathogens related to upper respiratory infections (URIs) [1,2]. With more than 80% of children experiencing OM during early childhood [3], the financial burden of OM is estimated to be around $4.3 billion (USD) annually in the United States [4]. The diagnosis of OM is established when a middle ear effusion is present in a normally aerated middle ear cavity, which is the space behind the eardrum [5,6,7]. When the presence of a middle ear effusion persists for several months, OM may lead to conductive hearing loss, causing speech and language delays for young patients [7]. The burden of OM is more substantial in developing countries as with other infectious diseases [8].

Despite a high prevalence of OM, the current diagnostic methods suffer from the limited capability of the standard diagnostic tool, an otoscope. Physicians can examine the eardrum, or the tympanic membrane (TM), and the space behind the TM via otoscopy if the TM is translucent. However, it is highly challenging to visualize an effusion in the middle ear cavity during OM due to the inflamed and opaque TM. As a result, the diagnostic accuracy of standard otoscopy alone ranges from 46–74%, depending on physician expertise and experience [9].

To overcome the diagnostic challenges of conventional otoscopy, optical coherence tomography (OCT) has been implemented in probe-based, portable systems for middle ear imaging [10,11,12,13,14]. Other form factors or beam-delivery systems, such as endoscope- and catheter-based OCT systems, have also been investigated for imaging the middle ear in vivo [15,16] and the Eustachian tube ex vivo [17,18]. OCT, first developed in 1991 [19], provides depth-resolved, cross-sectional images similar to ultrasound imaging, though employing near-infrared light instead of acoustic waves. OCT collects backscattered light from the TM and up to 2–3 mm inside the middle ear cavity, generating a depth-resolved structural map based on differences in refractive indices. Many previous studies have shown that OCT can provide quantitative information on different middle ear conditions during OM, including the presence, amount, and rough viscosity of middle ear effusions [20,21,22]. With cellular-level resolution (2–10 µm in depth), OCT can detect the presence of a middle ear biofilm adherent to the TM [23,24,25], which plays a major role in recurrent and chronic OM [26,27,28,29]. Biofilms are aggregated bacteria encased in an extracellular matrix, often adhered to the inner surface of the entire middle ear cavity during OM, and confer greater antibiotic resistance [27,28]. In comparison, standard otoscopy or any other non-invasive method, cannot accurately detect the presence of biofilm in the middle ear.

OCT is a new promising technique for otology [14,30] primarily because OCT non-invasively provides quantitative diagnostic insights to detect and assess effusions and biofilms during OM. There have been several key challenges for further translation of the technology. First, portable OCT systems have historically been bulky and expensive compared to the standard middle ear diagnostic tools, as a complete system contains a light source, a detector, associated optics, a computer or processing unit and a screen. Recently, a compact, low-cost OCT system (~$8000 USD) with a reasonable system performance (an axial resolution of 8 µm with a 10 kHz A-scan rate) was developed [31]. Due to the design goals of the imaging system to reduce cost, it had a limited imaging depth and optical power that made difficult to quickly focus the light on a thin TM using a handheld probe in clinical settings. Second, clinicians are not familiar with acquiring and interpreting OCT images of the middle ear. The OCT technology would be more efficiently translated to clinics with an automated interpretation of images, as the utility of machine learning (ML) has been extensively investigated in various biomedical imaging and data [32,33,34]. Our previous study utilized the database of middle ear OCT images acquired from human subjects to develop a ML algorithm based on a random forest classifier [35]. An overall accuracy of 91.5% was achieved when compared with physician diagnosis, highlighting the impact of quantitative, visualized information inside the middle ear.

In this paper, we present a ML classifier-integrated compact briefcase imaging system for translational middle ear imaging. The real-time ML classifier enhances the capability of this briefcase imaging system as a stand-alone, portable diagnostic device that does not require prior knowledge of OCT or OM. To demonstrate this, the users with different proficiency levels with OCT and ear imaging were trained to operate the briefcase system and the results were correlated. Finally, representative datasets from outpatient subjects clinically diagnosed with OM are presented, demonstrating the diagnostic capability of this briefcase device. An automated, on-site interpretation of OCT middle ear images in a compact device will allow any user, including clinicians, nurses and parents, to monitor the middle ear conditions during OM. With continued growth and adoption of telehealth using compact diagnostic devices and smartphones assisted with artificial intelligence (AI), our system may improve remote examination, diagnosis, and treatment monitoring of OM.

## 2. Materials and Methods

### 2.1. Development of Compact Briefcase System for Middle Ear Imaging

**Figure 1:**
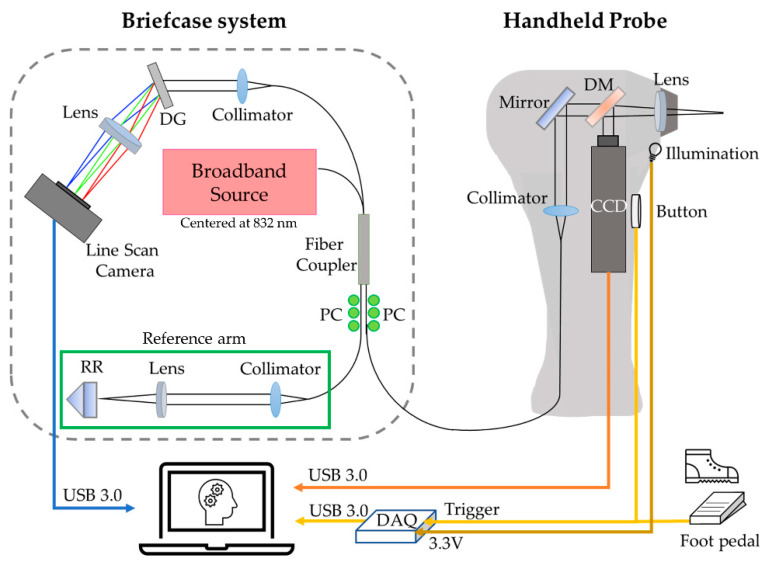
A schematic diagram of the ML-integrated, handheld briefcase OCT system. Data acquisition and processing are performed using a standard laptop. DG: diffraction grating; PC: polarization controller; RR: retroreflector; DAQ: data acquisition system; DM: dichroic mirror to spectrally separate light; CCD: charge-coupled device for simultaneous otoscopy.

A high-resolution OCT system for translational middle ear imaging was developed, based upon a previously developed low-cost briefcase system [31]. A schematic diagram of the briefcase system is shown in Figure 1. A superluminescent diode (SLD-351-HP2, Superlum, Carrigtwohill, Ireland) centered at 832 nm with a full-width-half-maximum (FWHM) bandwidth of 75 nm was used as a broadband light source. A compact, USB-based line-scan spectrometer (Compact Cobra-S, Wasatch Photonics, Morrisville, NC, USA) was used as a detector (2048 pixels, 12-bit), operated at a 20 kHz A-scan rate. An axial resolution was measured around 4.9 µm in air, with an imaging range of around 2.9 mm. A near-infrared achromatic lens (Edmund Optics, Barrington, NJ, USA) with a focal length of 60 mm and a diameter of 12 mm was used as an objective lens. The power incident on the sample was approximately 5.5 mW. The maximum sensitivity measured with a mirror was approximately 116 dB. A pseudo-cross-sectional image was obtained by manually moving or angling the probe, as this design did not require computer-controlled lateral scanning, which reduced complexity, size, weight and cost.

A low-cost, USB-based multifunction DAQ (USB-6003, National Instruments, Austin, TX, USA) was implemented to trigger an acquisition and to provide a voltage (3.5 V) for a miniature halogen lamp (03100-U, Welch-Allyn, Skaneateles Falls, NY, USA) that was used to illuminate the ear canal. A simultaneous surface image of the TM was acquired through a compact CCD camera at 17 Hz (MU9PC-MH, XIMEA, Münster, Germany). All data acquisition and processing were performed by a laptop (HP OMEN, CE019DX, i7–7700HQ CPU, 8 GB RAM, Palo Alto, CA, USA) via a USB 3.0 connection using a custom-developed software platform, LabVIEW 2017SP (National Instruments, Austin, TX, USA) and MATLAB R2018a (MathWorks, Natick, MA, USA).

**Figure 2:**
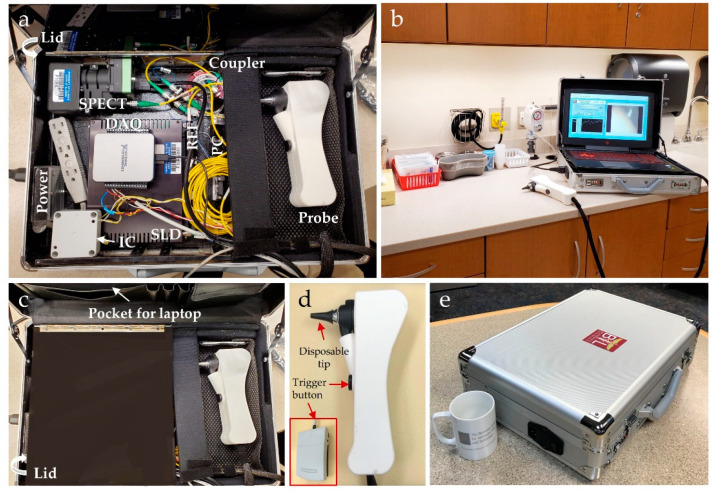
Photos of the complete ML-integrated briefcase system for ear imaging. (**a**) All components including associated optics, 3D-printed handheld probe and laptop fit into a standard briefcase; (**b**) briefcase system in an exam room for translational middle ear imaging; (**c**) a pocket stores the laptop during transport, and a lid protects the optical system from damage; (**d**) detailed view of the handheld probe with a disposable ear speculum and trigger buttons; (**e**) closed briefcase system with a standard coffee mug for size comparison. SPECT: spectrometer; IC: illumination circuit for a halogen lamp; REF: a reference arm that allows light to travel for a fixed distance in OCT; SLD: superluminescent diode; PC: polarization controller; DAQ: data acquisition system.

Photos of the complete ML-integrated briefcase system are illustrated in Figure 2. The OCT system, handheld probe, foot pedal, and laptop fit into a standard briefcase for transport (33 cm × 46 cm × 13 cm, height × width × depth). The entire system, including the laptop (2.3 kg), weighed around 9.1 kg. Figure 2d shows the handheld probe, with two buttons to trigger the ML classifier and data acquisition. A standard, disposable otoscope speculum (18c, RA Bock Diagnostics, Laramie, WY, USA) was used for each measurement.

### 2.2. Integration of Real-Time Machine Learning Classifier in the Briefcase System

A ML algorithm based on a random forest classifier was previously developed using OCT middle ear images acquired from human subjects (*n* = 25,479 A-scans, collected from 58 ears) [35]. To evaluate the performance of the classifier, “Leave-one subject (ear)-out” cross-validation (k = 58-fold) was performed to train, test, and validate the random forest classifier [35]. Furthermore, the random forest classifier showed the optimal performance compared to other models, such as ensemble, SVM, and kNN [35]. An overall accuracy of detecting the presence of middle ear contents was determined to be 91.50% compared to physician diagnosis [35]. Further details on developing the classifier model are described in Monroy et al. [35].

The OCT images trained in the ML classifier were collected from a custom-built high-end OCT imaging system [20] with a higher axial resolution (~2.4 µm in air) and a faster acquisition (~32 kHz of A-scan rate) than the briefcase system, with a lateral scanning (field-of-view of ~4 mm). However, Monroy et al. [35] also demonstrated that OCT middle ear images with a sufficient signal-to-noise ratio (a SNR of ~80 dB) and a resolution (an axial resolution of 19.2 µm or better) can still provide accurate classification using the developed ML algorithm. This allowed a direct implementation of the previously developed ML classifier to the compact briefcase middle ear imaging system in this study.

In this study, the compact, high-resolution briefcase system was implemented with the previously developed ML classifier in real-time. Note that only A-scan-based features were utilized in the classifier for the briefcase system. The extracted features of each A-scan include an axial thickness of the TM, peaks, and attenuation profiles. An axial thickness of the TM was calculated by detecting the two predominant peaks in each OCT A-scan. An increased thickness of a TM may indicate inflammation during the infection. In addition, each A-scan contained multiple peaks generated from optical scattering of tissue, and the number of the peaks was determined for each A-scan. The greater number of the detected peaks may indicate the presence of middle ear contents that scatter light, compared to the empty middle ear. Lastly, an estimated attenuation coefficient was calculated [36], as each A-scan contains information of optical attenuation in tissue. The higher optical attenuation may indicate the greater density of middle ear contents.

**Figure 3:**
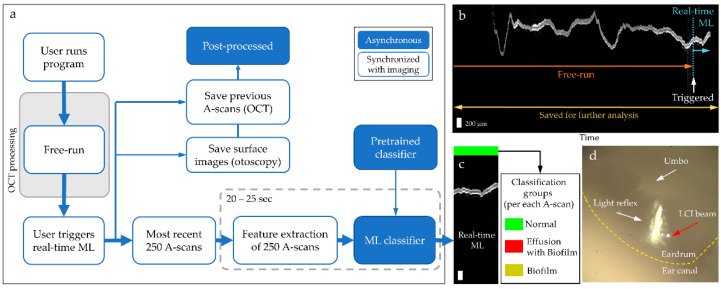
Processing flow and representative output of the ML-integrated briefcase system. (**a**) Step-by-step illustration of image acquisition, processing and feeding to the ML classifier; (**b**) representative OCT image showing complete dataset: during free-run and the post-triggered data (250 A-scans) sent to the real-time ML classifier; (**c**) output display of the classifier; (**d**) simultaneously acquired surface image of the TM labeled with major anatomical landmarks.

The processing flow of the ML-integrated briefcase system is illustrated in Figure 3a. Figure 3b shows a representative dataset acquired from the briefcase system. Note that the system first starts with free-run mode, where the user navigates the ear canal and positions the beam over the region of interest, such as the light reflex (cone of light) on the TM. Once the light is focused and stabilized, the user triggers the ML classifier and data acquisition using either the button on the handheld probe or the foot pedal. A subset of the post-triggered data is automatically processed for the feature extraction, and then classified. The results are displayed after ~20–25 s. The entire data scan is saved for later processing.

In Figure 3c, the displayed classifier output in real-time is derived from the most recent 250 A-scans, where each A-scan is classified as either normal, ear with effusion and biofilm or ear with biofilm. As each A-scan is classified as one of the three groups, a line classification (0–100%) of three groups can be represented for each image compiled of the acquired A-scans. Demonstration of acquiring a middle ear OCT image and its ML classification included in Supplementary Information Video S1. Figure 3d shows a simultaneously taken surface image of the TM and light reflex, where a red arrow indicates the focused OCT beam from the briefcase system.

### 2.3. Imaging Human Subjects

This study was conducted under a protocol approved by the Institutional Review Boards at the University of Illinois at Urbana-Champaign and Carle Foundation Hospital in Urbana, Illinois. Informed consent was collected from each recruited human subject. In the first part of the study, a total of four adult volunteers (8 ears) with normal middle ear conditions participated to investigate the user-variability in operating the briefcase system. All volunteers did not exhibit any ear-related symptoms in the past year prior to imaging, and normal middle ear conditions were confirmed by ‘A type’ tympanograms from a commercial tympanometer (AutoTymp TM286, Welch-Allyn, Skaneateles Falls, NY, USA). Tympanometry is one of the standard middle ear diagnostic tools which detects the presence of middle ear effusions by measuring the acoustic compliance of the TM. In addition, OCT images of the middle ear were acquired from the volunteers using both the briefcase system and a high-end portable OCT system [20] for comparison. Standard video otoscopy (Digital MacroView, Welch-Allyn, Skaneateles Falls, NY, USA) was performed to collect a high-resolution image of the TM.

All volunteers were trained for 1 h prior to operating the briefcase system. The users (volunteers) were instructed to focus the light near the light reflex (cone of light) as depicted in the screen by simultaneous surface visualization of the TM. Then, the users imaged each other’s ears to compare the acquired images and classified results between the users. Among the four users, two were considered as OCT and otoscopy imaging experts with more than 5 years of experience, while two had no prior experience with OCT or otology. The users obtained around 2–4 measurements per ear, in which each measurement took around 1–3 min.

Next, two adult subjects clinically diagnosed with OM were recruited from the Carle Health outpatient Ear, Nose, and Throat (ENT) clinic with an appropriate consent procedure. Imaging was performed in a standard exam room at Carle Foundation Hospital in a busy clinical environment (Figure 2b). A total time duration for each subject was 20–30 min, which included consenting, imaging of both ears by three trained users who have different levels of experience in OCT and otology (ENT physician, OCT expert, and research coordinator), and waiting time between each user. The de-identified examination reports were collected after the study.

### 2.4. Analytical Methods

Throughout the study, a line classification (%) was calculated using the outputs from individual A-scans to evaluate the ML classifier. For example, if all A-scans were classified as ‘Normal’, the results would be interpreted as ‘Normal’, with a ‘Normal’ line classification of 100%. When post-processing the entire dataset collected, A-scans without any optical signals during the free-run were excluded to calculate the line classification. For the first part of the study with healthy volunteers, the thickness of the TM was also computed to compare OCT images acquired from the briefcase system and from the high-end system. OCT images were converted into binary masks followed by a median filtering, thresholding, and segmentation. The thickness of the TM was computed, assuming a refractive index of the TM of 1.44 [37].

The line classification between the Expert and Novice user groups were compared using a two tailed Student’s t-test to determine whether the results were independent of the users’ experiences. The thickness of the TM measured from the two different systems as well as from three individual users of the briefcase system were statistically evaluated using a two-way analysis of variance (ANOVA) with a multiple comparison test. All statistical analysis was performed in MATLAB R2019b (MathWorks, Natick, MA, USA).

## 3. Results

### 3.1. Middle Ear Imaging of Healthy Volunteers

The briefcase system captures pseudo-cross-sectional images of the human TM in vivo, with a deeper imaging depth, an increased power and an improved speed compared to the previously developed low-cost briefcase system [31]. Overall, the ML-integrated briefcase system was approximately three times more expensive (~$24,000 USD) than the previously developed low-cost briefcase system, but was still approximately half the cost of the high-end OCT system.

**Figure 4:**
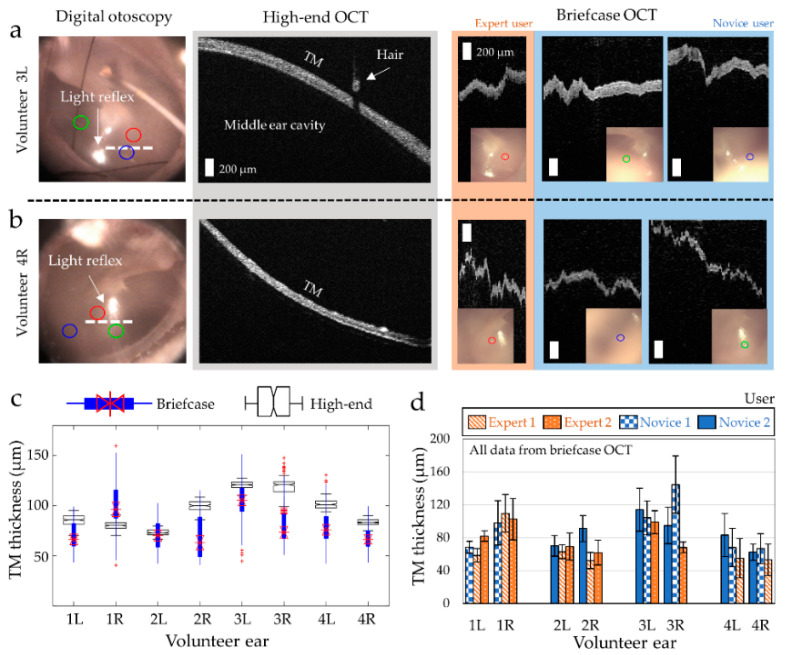
Side-by-side comparisons of middle ear images acquired from the high-end OCT system and the briefcase system. (**a**,**b**) Representative high-resolution digital otoscopy (white dotted line—the scanning region of the high-end OCT; circles—the focus of the briefcase OCT from the three different users), high-end OCT images and briefcase OCT images of the healthy middle ear; (**c**) overlaid box plots of the TM thickness measured from the briefcase (blue) and the high-end OCT system (black); (**d**) bar graphs comparing the user variability in TM thickness measured from the briefcase system. Each statistical comparison is shown in Supplementary Information Table S1.

In the first part of the study, middle ear OCT images of healthy adult volunteers were acquired using both the briefcase system and the high-end OCT system, which was used to generate the dataset for the classifier in the briefcase system [35]. Note that normal middle ear conditions of the volunteers were confirmed with ‘Type A’ tympanogram. Representative high-resolution OCT images of healthy middle ears are shown in Figure 4a,b. The white dotted lines in otoscopy indicate the scanning region of the high-end OCT system. It was observed that the thickness of the TM in Volunteer Subject 3L (Figure 4a) was around 41% thicker than that of Volunteer Subject 4R (Figure 4b). The same healthy volunteers were again imaged with the briefcase system by three trained users. Depending on the level of prior experience with OCT and otoscopy, the users were divided into Expert and Novice groups. The focus of the beam is shown in red, green, and blue circles, and their locations are also visualized with high-end digital otoscopy in Figure 4a,b. Throughout the study, the users were instructed to obtain measurements near the light reflex to minimize the spatial dependence of variations that are found across the TM. The difference in TM thickness between Volunteer Subjects 3L and 4R was also noticeable from the briefcase OCT measurements, as expected. Since the briefcase system was not implemented with a scanning mechanism, the horizontal axis of the briefcase image corresponds to time, rather than space, whereas the horizontal axis in the high-end OCT image represents the lateral spatial location. Analogous to ultrasound imaging, the briefcase system generates M-mode images (depth at one location vs. time), whereas the high-end OCT system generates B-mode images (depth vs. lateral location).

The thickness of the TM in four healthy adult volunteers is compared in Figure 4c. The normal human TM in adults can range from 50 µm to 120 µm [37], depending on the region over the TM. It is worthwhile to mention that the measurements from the high-end system were obtained from a lateral scanning (white dotted lines in Figure 4a,b), whereas the measurements from the briefcase system were obtained from point-based measurements in three distinct regions acquired from three different users. Therefore, a greater variability in the TM thickness from the briefcase measurements was observed, in which the variability between users is separately assessed in Figure 4d. Even though most measurements within the same ear largely overlap (i.e., 1R, 2L, 3L, and 4L), greater differences were observed from one user imaging the Volunteer Subject 2R and another user imaging the Volunteer Subject 3R (Figure 4d) and both users were Novice users. The *p*-value of each comparison between users per ear is included in Supplementary Information Table S1. Statistically significant differences were found from the spatially different measurements confined to a very narrow point on the TM. Nonetheless, the results suggest that the compact briefcase system can be used to capture optical ranging data from the human middle ear, quantify the thickness of the TM and be operated by trained users regardless of their prior knowledge in OCT or otology.

### 3.2. User-Invariant Classifications of Healthy Middle Ear

**Figure 5:**
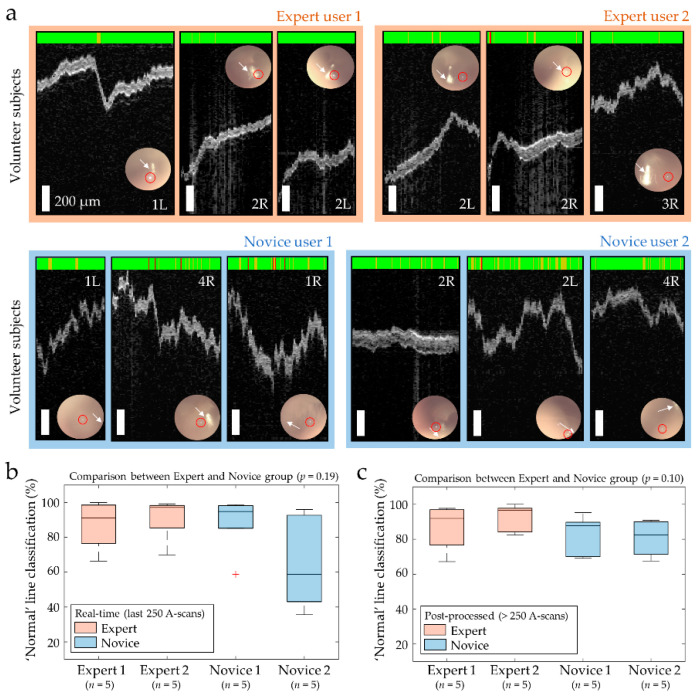
ML classified results between users with different levels of experience. (**a**) Representative briefcase images of three healthy ears with simultaneous otoscopy (inset; red circle and white arrow indicate the focusing beam and light reflex, respectively) and the ML-classified results on the top bar; (**b**) box plot of the real-time classification results during imaging; (**c**) box plot of the classification results after post-processing the entire dataset.

Representative ML-classified images from the first part of the study with healthy adult volunteers are shown in Figure 5a. The colored bar on top indicates the outputs from the ML classifier, for each A-scan (column). It is expected that all healthy volunteers will result in ‘Normal’ middle ears. A few ‘Abnormal’ A-scans appeared with abrupt movements of the subject or the handheld probe, strong light reflections, or image artifacts. The four trained users with different levels of experiences imaged each other’s ears, which were confirmed as normal middle ears. As shown by red circles (focused beam) and white arrows (light reflex) in Figure 5a, the Expert users tended to focus the beam closer to the light reflex than the Novice users did. In addition, the Expert users who were familiar with OCT and otoscopy generally collected more stable images, as expected. Nevertheless, the ML classification consistently and accurately resulted in a ‘Normal’ classification regardless of the user. These results also validate that the ML algorithm can generate accurate classifications of the middle ear even if OCT images were acquired by a different OCT system that was used to generate the training dataset, if the SNR and axial resolution are reasonable (a SNR greater than ~80 dB with an axial resolution of 19.2 µm or better) [35].

Next, a line classification was evaluated between the users. First, the real-time ML classifications during the image acquisition were compared for each user and are plotted in Figure 5b. There was no statistical difference in the ‘Normal’ line classification between the Expert users (2 users, 10 total measurements) and the Novice users (2 users, 10 total measurements), with *p* = 0.19. Figure 5c compares the ‘Normal’ line classification after post-processing the entire dataset collected. Even though the line classification of the dataset collected by the Expert users was slightly higher than that of the Novice users, there was no statistical difference, with *p* = 0.10. The *p*-value was slightly lower in the latter case because the Novice users tended to generate more abrupt movements and image artifacts throughout the entire dataset than the Expert users, who were more familiar with OCT and clinical ear imaging of human subjects.

### 3.3. Machine Learning Classification of Middle Ear Conditions during OM

**Figure 6:**
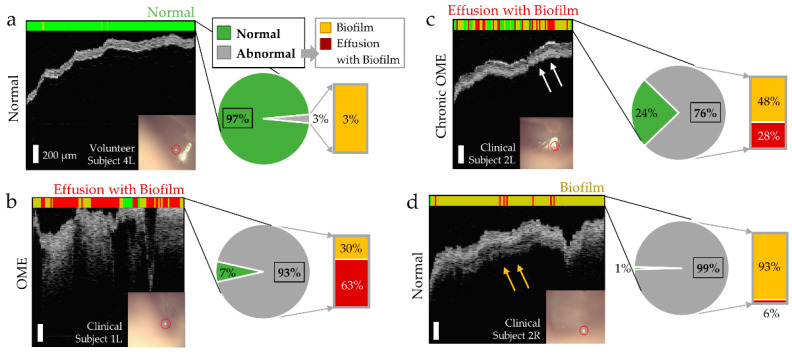
ML-briefcase OCT measurements from subjects clinically diagnosed with OM. (**a**) Representative results from a normal middle ear for comparison; (**b**) results from a subject diagnosed with OME, where most regions were classified as ‘Abnormal’ (containing effusion with biofilm); (**c**) results from a subject in which her left ear was diagnosed with chronic OME, and OCT detected additional structures behind the TM (white arrows); (**d**) results of the right ear from the same subject as in (**c**), and the additional structures are visualized (yellow arrows). However, note that this ear was clinically diagnosed with only an otoscope as being a normal middle ear. Red circles in (**a**–**d**) indicate the location and focus of the OCT beam.

To assess its clinical significance, the ML-integrated briefcase imaging system was transported to a local hospital (Carle Foundation Hospital, Urbana, IL, USA). Subjects clinically diagnosed with OM participated in the study. The ML-classified results comparing the normal middle ear and the ears diagnosed with OM are shown in Figure 6. Here, a pie chart is provided to visualize the line classification. Figure 6a indicates the empty middle ear space, clear boundaries of the TM, and homogeneous and consistent thickness of the TM, all indicating the signs of normal middle ear. Figure 6b shows the image acquired from the Clinical Subject 1 diagnosed with otitis media with effusion (OME). The ML algorithm resulted in ‘Abnormal’, with 93% of the ‘Abnormal’ line classification. The classifier also suggested that this ear contained effusion with biofilm. Figure 6c shows another scenario, in which a subject was diagnosed with chronic OME. Even though the middle ear space was mostly clear from the OCT image, OCT detected an additional layer adhered to the TM (white arrows). The ML classifier resulted in ‘Abnormal’, with 76% of ‘Abnormal’ line classification. The classifier suggested that this ear likely contained an effusion with biofilm.

Note that only the left ear of Clinical Subject 2 was clinically diagnosed with OM, and the right ear was clinically diagnosed as normal, using a standard otoscope. However, OCT detected additional structures behind the TM (yellow arrows in Figure 6d) from the right ear of Clinical Subject 2. As a result, the right ear of Clinical Subject 2 was classified as ‘Abnormal’ and suggested that this ear contained a middle ear biofilm. This is not surprising considering that this subject’s left ear was diagnosed with chronic OME, and OM often occurs bilaterally in both ears. However, the ML classifier did not indicate that this ear contained an effusion, which agreed with physician diagnosis.

The datasets shown in Figure 6a,c,d were collected by an Expert user, while the dataset shown in Figure 6b was collected by the Novice user (research coordinator) who did not have any prior experience with OCT and otoscopy imaging. Even though three users with different levels of experience (researcher experienced in OCT ear imaging, ENT physician and research coordinator) imaged three ears from the two clinical subjects, three classified results were obtained from one ear (Clinical Subject 2R) out of three ears. This was largely due the time constraints and the limited number of imaging attempts (1–3 attempts for each ear), which resulted in greater motion artifacts, flipped OCT images and low SNR. Nonetheless, Clinical Subject 2R was classified as ‘Abnormal with Biofilm only’ from all three users.

## 4. Discussion

The adoption of telemedicine has gradually increased over the past decades in the United States [38] and particularly during the coronavirus (COVID-19) pandemic [39]. In otolaryngology, telemedicine may involve low-cost, compact, diagnostic devices or smartphones, which enable remote examination and monitoring of various diseases [40]. With continuous advances in AI techniques and big data, recent investigations have developed AI-assisted imaging platforms for otoscopy to diagnose and monitor OM [41,42,43]. Several studies have shown that by using a ML approach on otoscopy images for detecting middle ear effusions, an overall accuracy of 84–94% can be achieved [43,44,45], compared to an overall diagnostic accuracy of 50–70% by physicians with standard otoscopy alone [46]. However, although the AI algorithms may detect subtle features in the surface otoscopy images, they still suffer from limited information about the content of the middle ear cavity (air, effusion and biofilm) because the images used to train the AI algorithms provide only surface information on the TM.

This study presents a ML-integrated compact briefcase system to provide and interpret OCT images of the middle ear in a clinical setting. Depth-resolved OCT signals provide visualization and quantitative information inside the middle ear cavity. The compact system in the present study offers an axial resolution of ~4.9 µm in air, an imaging range of ~2.9 mm and an incident optical power of 5.5 mW, all in a briefcase form factor (Figure 2) enabling middle ear imaging within a minute (Video S1). Furthermore, the real-time ML classifier in the briefcase system can assist with the diagnostic classification of the middle ear OCT images (Normal, Biofilm, and Effusion with Biofilm) on site, compensating for the image quality and the lack of a lateral scanning mechanism compared to the high-end OCT system. Note that comparing the performance of this work with previous ML models with otoscopy alone is not directly possible, because otoscopy does not provide cross-sectional images. OCT provides new and superior information about the content of the middle ear, behind the TM and where the effusion and infection lies.

More importantly, users with no prior knowledge of OCT and otoscopy imaging were trained to operate the system and collected useful data. In general, all users took around 1–3 min to obtain one measurement when imaging healthy adult volunteers, after 1 h of training. The ‘Normal’ line classification achieved by the Novice users were not statistically different from those acquired by the Expert OCT users (Figure 5), suggesting a great potential as a stand-alone, user-invariant diagnostic imaging device. The users can acquire more stable images by using the foot pedal instead of pressing the button on the probe, which can generate greater hand motions. It is also expected that with more experience in operating the device, the Novice users will learn to acquire more stable measurements.

To assess the clinical significance of the ML-integrated briefcase system, subjects diagnosed with OM were also recruited in this study. OCT detected and visualized the additional scattering structures behind the TM as well as a greater thickness of the TM from the subjects with OM, when the ear was clinically diagnosed with OM (Figure 6b,c). In addition, a thin, additional layer adherent to the TM was also visualized from our briefcase imaging system, which was not obvious with standard otoscopy. The ML classifier correctly generated an abnormal interpretation, with a prediction of what the additional structures were likely to be (effusion and/or biofilm). However, one ear (Subject 2, Left) received an abnormal classification from the briefcase system, whereas that ear was clinically diagnosed as normal by the physician (Figure 6d). The classifier did not indicate that this ear contained an effusion, but only biofilm. This agreed with the physician’s diagnosis that the middle ear was clear. However, the presence of the biofilm was not surprising given the patient’s chronic OME history. These findings suggest that the depth-resolved optical information from the briefcase system may provide complementary and possibly even superior diagnostic information, compared to standard otoscopy alone.

There are several limitations to be discussed. In general, the processing power and memory of the laptop were limiting factors in the speed of OCT processing and display, as well as for the ML classifier. Note that all the hardware and optical components used in the system were off-the-shelf products. Implementing graphic processing units (GPUs) will further accelerate the speed of the system. With emerging technologies and products in compact OCT waveguides that integrate a light source and detector [47,48], the size of the system can be further reduced in the future. As the briefcase system did not utilize a lateral scanning element to generate B-mode images of the middle ear, the measurements can be affected by the spatial locations of the focused beam on the TM. Implementing a compact lateral scanning mechanism using a microelectromechanical systems (MEMS)-based mirror can reliably provide lateral information, which will help minimize the spatial dependence of the measurements. This may further improve the usability and system performance.

In the future, with a larger database and an improved model, hyperparameter tuning will be performed to compare different ML models. This model has been internally validated and evaluated using the leave-one-out cross-validation method. External validation was limited due to the small size of the dataset. With the increasing number of ear OCT images and datasets in the future, the model will be further improved by external validation using a held-out, independent dataset.

The spatial dependence of measurements from the TM can also be overcome with an improved ML classifier. For example, the dataset of OCT images containing different regions on the TM can be obtained and trained in the ML classifier. OCT images with various image artifacts (mirror artifacts, flipped image and strong reflections) can also be collected and included in the training dataset. This will exclude the measurements with artifacts and can potentially guide the users to avoid these artifacts during imaging. It was also observed that the Novice users heavily relied on the surface images of the TM to guide and focus the laser beam during the imaging. In the future, a CCD camera with a higher resolution, a larger field-of-view and a greater depth-of-focus to capture the entire TM will be helpful for users without prior knowledge of OCT. The surface images of the TM were not included in the classifier, as the properties of the CCD images (i.e., lighting, field-of-view, depth-of-focus and resolution) from the briefcase system were different from the trained otoscopy images in the previously developed classifier. With greater computing power in the future, providing the surface images of the TM to the classifier may enhance the classification accuracy.

Finally, having rigid time constraints to image patients’ ears in a busy clinical environment may have resulted in suboptimal image quality in some cases. While trained users without prior experience of OCT and otoscopy attempted to image the subjects diagnosed with OM, not all users were successful to acquire reliable datasets because of the given time constraints. Allowing for a longer imaging time window with more training and practice will improve image quality. A larger number of subjects diagnosed with OM will be necessary in future clinical studies to better characterize the clinical significance and accuracy of the system. In addition, this study only recruited adult subjects because the subjects needed to be tolerant to allow three different users to image their ears. A future study will include pediatric subjects as well and investigate differences that may emerge in this patient population. Lastly, more investigations are necessary to correlate and evaluate the dataset, ground truth, and the label, to determine the diagnostic importance of AI-assisted OCT otoscopy in clinical practice [35].

## 5. Conclusions

A ML-integrated compact briefcase OCT system has been developed to improve the portability of the imaging device and to provide ML-assisted interpretation of middle ear OCT images for detecting and diagnosing OM. Users with no prior knowledge of OCT and middle ear imaging were trained to operate the system. The classifier outputs suggested that Novice users can collect reliable middle ear OCT images, and receive classification results that are not statistically different (*p =* 0.19) from the results obtained from Expert users. The ‘Abnormal’ classifications with information on the presence of middle ear effusions and biofilms were achieved from subjects clinically diagnosed with OM, emphasizing the potential of the ML-interpreted OCT imaging in clinical applications. The ML-integrated OCT system may provide user-invariant interpretation of middle ear conditions, which can improve overall detection, diagnosis, monitoring and management of OM in remote settings.

## Data Availability

The data that support the findings of this study are available upon reasonable request to the corresponding author and under a collaborative research agreement.

## Supplementary Materials

The following are available online at https://www.mdpi.com/article/10.3390/bios11050143/s1, Table S1: Results of a two-way analysis of variance (ANOVA) and a multiple comparison test comparing TM thickness measured by different users of the briefcase system, Video S1: Demonstration of middle ear imaging by the briefcase system.
