# Supplementary material for: Handheld Briefcase Optical Coherence Tomography with Real-Time Machine Learning Classifier for Middle Ear Infections
Source: Biosensors (Basel). 2021 May 3;11(5):143. doi: 10.3390/bios11050143 (PMC8147830; doi:10.3390/bios11050143)
Supplement: Supplementary file 1 [file biosensors-11-00143-s001.zip › ML-briefcase_supplementary_v2.5.pdf]

biosensors

MDPI

Supplementary Information

Handheld briefcase optical coherence tomography with real-time machine learning classifier for middle ear infections

Jungeun Won [1,2], Guillermo L. Monroy [2], Roshan I. Dsouza [2], Darold R. Spillman, Jr. [2], Jonathan McJunkin [3,4], Ryan G. Porter [3,4], Jindou Shi [2,5], Edita Aksamitiene [2], MaryEllen Sherwood [6], Lindsay Stiger [6], and Stephen A. Boppart [1,2,4,5,*]

[1] Department of Bioengineering, University of Illinois Urbana-Champaign, Urbana, IL
[2] Beckman Institute for Advanced Science and Technology, University of Illinois Urbana-Champaign, Urbana, IL
[3] Department of Otolaryngology, Carle Foundation Hospital, Urbana, IL
[4] Carle Illinois College of Medicine, University of Illinois Urbana-Champaign, Champaign, IL
[5] Department of Electrical and Computer Engineering, University of Illinois Urbana-Champaign, Urbana, IL
[6] Stephens Family Clinical Research Institute, Carle Foundation Hospital, Champaign, IL
* Correspondence: boppart@illinois.edu

1. Supplementary Table

Table S1. Results of a two-way analysis of variance (ANOVA) and a multiple comparison test comparing TM thickness measured by different users of the briefcase system in **Figure 4(f).**

**Subject ear**	Novice 1 – Expert 1	Expert 1 – Expert 2	Novice 1 – Expert 2
1L	$\boldsymbol{p < 0.001}$	$\boldsymbol{p < 0.001}$	$\boldsymbol{p < 0.001}$
1R	$\boldsymbol{p = 0.0022}$	$p = 0.3512$	$p = 0.6417$
	Novice 2 – Expert 1	Expert 1 – Expert 2	Novice 2 – Expert 2
2L	$\boldsymbol{p = 0.0033}$	$\boldsymbol{p = 0.0186}$	$p = 0.8911$
2R	$\boldsymbol{p < 0.001}$	$\boldsymbol{p < 0.001}$	$\boldsymbol{p < 0.001}$
	Novice 1 – Novice 2	Novice 1 – Expert 2	Novice 2 – Expert 2
3L	$\boldsymbol{p < 0.001}$	$p = 0.1469$	$\boldsymbol{p < 0.001}$
3R	$\boldsymbol{p < 0.001}$	$\boldsymbol{p < 0.001}$	$\boldsymbol{p < 0.001}$
	Novice 1 – Novice 2	Novice 1 – Expert 1	Novice 2 – Expert 1
4L	$p = 0.9999$	$p = 0.1199$	$p = 0.0763$
4R	$\boldsymbol{p < 0.001}$	$\boldsymbol{p = 0.0051}$	$p = 0.7172$

2. Supplementary Video (Video S1)

Video S1. Demonstration of middle ear imaging by the ML-briefcase system. The entire process from starting the program, locating the eardrum during free run, triggering the classification and data acquisition, and displaying the classified results is shown. The flow diagram is illustrated in **Figure 3**.
